# Longitudinal Whole-Exome Sequencing of Cell-Free DNA Reveals Molecular Evolution and Heterogeneous Clinical Outcomes in PD-L1 Stratified Advanced NSCLC Adenocarcinoma Patients Treated with Atezolizumab

**DOI:** 10.3390/ijms27072947

**Published:** 2026-03-24

**Authors:** Viola Bianca Serio, Tommaso Regoli, Debora Maffeo, Ignazio Martellucci, Diletta Rosati, Marco Ghisalberti, Alberto Balistreri, Gianluca Santamaria, Niccolò Vono, Francesca Mari, Francesca Colombo, Elisa Frullanti, Maria Palmieri

**Affiliations:** 1 Cancer Genomics & Systems Biology Lab, University of Siena, 53100 Siena, Italy; viola.serio@dbm.unisi.it (V.B.S.); tommaso.regoli@student.unisi.it (T.R.); debora.maffeo@dbm.unisi.it (D.M.); diletta.rosati2@unisi.it (D.R.); maria.palmieri@dbm.unisi.it (M.P.); 2 Med Biotech Hub and Competence Centre, Department of Medical Biotechnologies, University of Siena, 53100 Siena, Italy; 3 Department of Medical, Surgical and Neurological Sciences, University of Siena, 53100 Siena, Italy; francesca.mari@unisi.it; 4 Oncology Unit, Azienda Ospedaliero Universitaria Senese, 53100 Siena, Italy; ignazio.martellucci@ao-siena.toscana.it; 5 Thoracic Surgery Unit, Azienda Ospedaliera Universitaria Senese, 53100 Siena, Italy; marco.ghisalberti@ao-siena.toscana.it; 6 Bioengineering Laboratory, University of Siena, 53100 Siena, Italy; alberto.balistreri@dbm.unisi.it; 7 Department of Experimental and Clinical Medicine, University “Magna Graecia” of Catanzaro, Campus “S. Venuta”, Germaneto, 88100 Catanzaro, Italy; gsantamaria@unicz.it (G.S.); niccolo.vono@unicz.it (N.V.); 8 Clinical Pathology Unit, Azienda Ospedaliero-Universitaria Senese, 53100 Siena, Italy; 9 Institute for Biomedical Technologies, National Research Council, Segrate, 20054 Milan, Italy; francesca.colombo@cnr.it

**Keywords:** NSCLC, liquid biopsy, cfDNA, immunotherapy, atezolizumab, clonal evolution, molecular clearance, PD-L1, high-depth cfDNA WES, treatment response

## Abstract

Programmed death-ligand 1 (PD-L1) expression is routinely used to guide immune checkpoint inhibitor (ICI) therapy in advanced non-small cell lung cancer (NSCLC), yet clinical benefit remains heterogeneous even among PD-L1–high tumors. Liquid biopsy based on cell-free DNA (cfDNA) enables minimally invasive, real-time monitoring of tumor evolution. We report four cases of metastatic lung adenocarcinoma treated with atezolizumab, integrating longitudinal whole-exome sequencing (WES) of cfDNA with radiological assessment. Four patients with PD-L1–positive (≥60%) metastatic NSCLC received atezolizumab monotherapy. Serial cfDNA samples (1–3 per patient) were analyzed by high-depth WES. Distinct molecular trajectories paralleled divergent clinical outcomes. One patient achieved a complete molecular response, characterized by progressive clearance of *KRAS*, *ATM*, and *NF1* mutant clones, which was concordant with radiological remission. A second patient showed an initial molecular response, followed by clonal rebound of *TP53*, *NF1*, and *NOTCH2* mutant populations and the emergence of *PTEN* and *KIF1A* variants, suggesting clinical progression. Two patients exhibited primary resistance despite high PD-L1 expression, with persistent or expanding clones and early subclonal diversification; in one case, new *EGFR* and *BRAF* alterations emerged under treatment pressure. Notably, switching to platinum-based chemotherapy in a non-responder induced a measurable molecular response, highlighting discordance between PD-L1 status and immunotherapy efficacy. Longitudinal cfDNA WES captured dynamic clonal remodeling under immunotherapy and anticipated radiological outcomes. These findings underscore the clinical necessity of integrating dynamic molecular monitoring by liquid biopsy to overcome the limitations of static PD-L1 assessment, refine therapeutic stratification, and identify early resistance mechanisms in advanced NSCLC.

## 1. Introduction

Lung cancer (LC) develops from cells within the respiratory system, including the bronchi, bronchioles, and alveoli. It usually presents as a tumor mass that can obstruct airflow or cause bleeding in the lungs or bronchi. LC remains the leading cause of cancer-related deaths worldwide, with its high incidence and frequently poor prognosis posing a significant public health concern. Current estimates indicate that lung cancer is responsible for roughly 2.5 million new diagnoses and 1.8 million deaths each year, accounting for nearly 18% of global cancer fatalities [1,2]. The elevated mortality rate is largely due to late-stage detection and the aggressive behavior of the disease. Incidence is rising in industrialized countries, driven by risk factors such as smoking, exposure to hazardous substances like asbestos and radon, and environmental air pollution. Smoking has been extensively linked to lung cancer, with heavy smokers facing a 60-fold higher risk than non-smokers [3].

Non-Small Cell Lung Carcinoma (NSCLC) accounts for approximately 85% of cases [4]. Among its histological subtypes, adenocarcinoma is the most prevalent, often characterized by an aggressive clinical course and a frequent diagnosis at advanced stages. Genetic characterization of LC is increasingly guiding personalized treatment approaches, offering hope for improved survival and more effective therapies. The molecular landscape of LC is highly diverse, and understanding key genetic alterations is essential for developing individualized treatment plans. The most frequently implicated driver genes include *EGFR*, *KRAS*, *ALK*, *BRAF*, and *ROS1* [5,6,7,8].

In addition to these main driver mutations, other emerging biomarkers are gaining clinical importance, including *PD-L1*, *MET*, *RET*, and *NTRK* alterations [9,10]. PD-L1, an immune checkpoint protein, allows tumors to evade immune surveillance; its overexpression can contribute to more aggressive disease and resistance to immunotherapy. However, PD-L1 expression is a snapshot of spatial and temporal expression that often fails to capture the tumor’s dynamic genomic landscape under therapeutic pressure.

Despite advances in targeted and immune-based therapies, early detection remains critical for improving outcomes. To capture this dynamic evolution, liquid biopsy has emerged as a pivotal tool for precision oncology. By analyzing cell-free DNA (cfDNA) and its tumor-derived component (ctDNA), it is possible to perform longitudinal monitoring via minimally invasive blood draws. This approach overcomes the limitations of static tissue biopsies, providing real-time insights into clonal evolution, treatment response, and the emergence of resistance mechanisms [11,12,13,14,15].

Here, we present four cases of advanced NSCLC adenocarcinoma with PD-L1 positivity, all treated with atezolizumab monotherapy. Despite their shared histological and therapeutic characteristics, these patients exhibited markedly divergent clinical trajectories. In this study, we used longitudinal whole-exome sequencing (WES) of cfDNA to decipher the molecular drift and clonal dynamics that underpin the heterogeneous clinical outcomes in a cohort of PD-L1-stratified NSCLC patients. Full methodological details are reported in the Supplementary Methods. The data are integrated with clinical assessment and imaging-based follow-up, highlighting the potential of cfDNA analysis to elucidate heterogeneity in treatment response in immunotherapy-treated lung cancer.

## 2. Case Presentation

Four patients (2 males and 2 females) diagnosed with advanced NSCLC adenocarcinoma were enrolled at the Oncology Unit of Azienda Ospedaliero-Universitaria Senese (AOUS). All patients exhibited PD-L1 expression levels >60% meeting the eligibility criteria for first-line atezolizumab monotherapy (Table 1).

At diagnosis (T0), peripheral blood samples were collected to perform cfDNA-based liquid biopsy, enabling baseline molecular characterization prior to treatment initiation. For longitudinal monitoring, serial blood samples were obtained at each follow-up time point to compare genomic profiles across multiple liquid biopsy assessments, along with the radiological images.

Notably, three distinct clinical response patterns to atezolizumab were observed among the patients. Despite shared PD-L1 positivity and identical treatment, the heterogeneous clinical and molecular trajectories revealed a profound decoupling between PD-L1 status and genomic evolution. These findings underscore the limitations of relying solely on PD-L1 expression as a predictive biomarker and emphasize the need for more refined, dynamic, and personalized molecular stratification strategies. The molecular profiles resulting from each cfDNA liquid biopsy for each patient are reported in the Supplementary Table S1.

### 2.1. First Case

**Figure 1:**
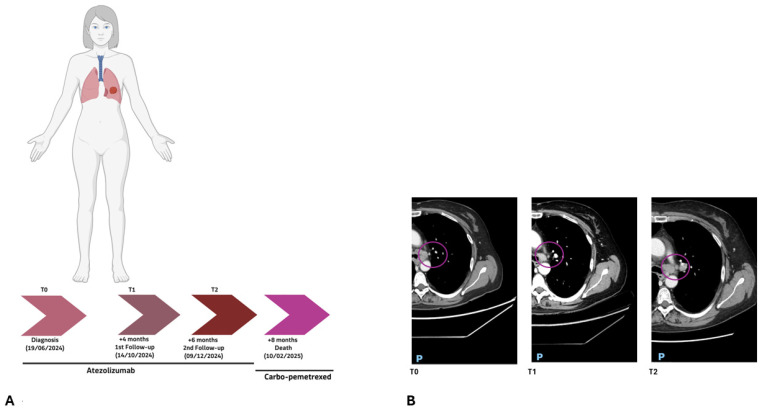
(**A**) 66/24 patient clinical timeline showing the NSCLC diagnosis, treatment with atezolizumab, and subsequent follow-ups. (**B**) CT scans at baseline (T0), first follow-up (T1), and second follow-up (T2), confirming a first response and subsequent lack of response to immunotherapy. The purple circle outlines the lung tumor mass. Anatomical orientations are indicated as P (posterior).

**Figure 2:**
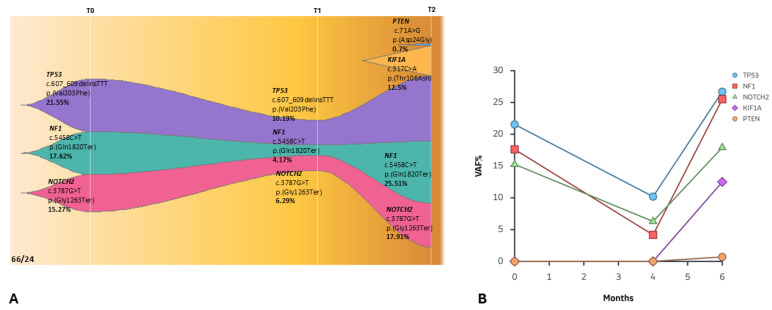
(**A**) Longitudinal monitoring of patient 66/24 under atezolizumab treatment. At T0 (baseline), three different expanded clones have been identified. At the first follow-up (T1), 4 months after, the cfDNA analysis showed a decrease in their VAF. Subsequently, after 2 more months, the previous clones were expanded again, gaining two new mutated clones in the *PTEN* and *KIF1A* genes. A different color represents each clone. (Source: ChrisMiller fishplot-R) (**B**) Clonal evolution plot illustrating the variant allele frequencies (VAFs) of somatic clones detected during longitudinal cfDNA monitoring (T0, T1, T2) under atezolizumab treatment.

Patient 66/24 (Figure 1A), a 55-year-old female (PD-L1: 60%), underwent baseline high-depth cfDNA WES at diagnosis (T0). Molecular profiling identified three predominant expanded clones involving pathogenic variants in *TP53* [c.607_609delinsTTT; p.(Val203Phe)], *NF1* [c.5458C>T; p.(Gln1820Ter)], and *NOTCH2* [c.3787G>T; p.(Gly1263Ter)], with variant allele frequencies (VAFs) of 21.55%, 17.62%, and 15.27%, respectively (Figure 2A,B).

The patient initiated first-line atezolizumab monotherapy. After four months of treatment (T1), a second cfDNA assessment demonstrated a marked reduction in the VAFs of the previously detected driver mutations, *TP53* [c.607_609delinsTTT; p.(Val203Phe)], *NF1* [c.5458C>T; p.(Gln1820Ter)], and *NOTCH2* [c.3787G>T; p.(Gly1263Ter)], which decreased up to 10.19%, 4.17%, and 6.29%, respectively (Figure 2A,B). This molecular response paralleled radiological findings, demonstrating a significant reduction in tumor burden. However, at the subsequent follow-up two months later (T2), a third cfDNA analysis revealed clear molecular progression (Figure 2A).

Specifically, a rapid increase in the VAFs of the previously identified driver clones (*TP53*, *NF1*, and *NOTCH2* reaching VAFs of 26.7%, 25.51%, and 17.91%) was observed, accompanied by the emergence of two novel variants in *KIF1A* [c.317C>A, p.(Thr106Asn), VAF = 12.50%] and in *PTEN* [c.71A>G, p.(Asp24Gly), VAF = 0.70%].

These findings were consistent with acquired resistance to atezolizumab and paralleled radiological evidence of disease progression (Figure 1B). At the second follow-up, cerebral metastasis was identified. Despite the therapeutic switch to carboplatin plus pemetrexed, the patient succumbed to the disease two months later.

### 2.2. Second Case

**Figure 3:**
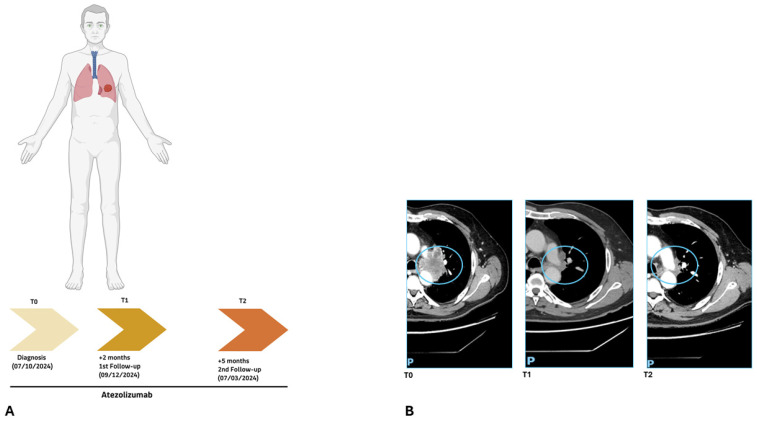
(**A**) 111/24 patient clinical timeline showing the NSCLC diagnosis, treatment with atezolizumab, and subsequent follow-ups. (**B**) 111/24 Patient CT scans at baseline (T0), at first follow-up (T1), and at second follow-up (T2), confirming a response to immunotherapy. The light-blue circle outlines the lung tumor mass. Anatomical orientations are indicated as P (posterior).

Patient 111/24, a 68-year-old male, presented with high PD-L1 expression (90%) and initiated first-line atezolizumab monotherapy (Figure 3A).

**Figure 4:**
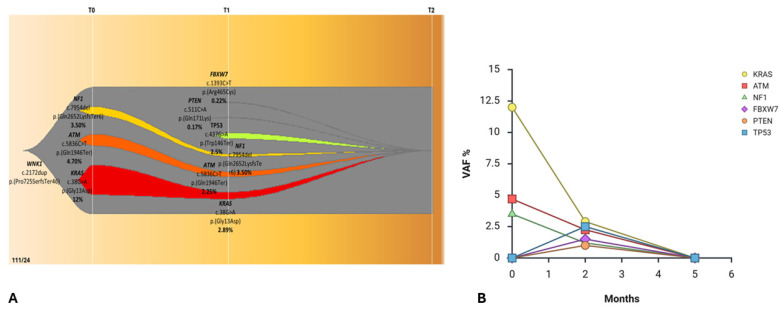
(**A**) 111/24 fishplot depicting the clonal architecture and dynamics inferred from the three-point liquid biopsy analysis. At the baseline (T0), three distinct expanded clones were identified. After 2 months (T1), cfDNA analysis showed a reduction in their VAFs, with the appearance of three new mutated clones in the *FBXW7*, *PTEN* and *TP53* genes. Three months later, we observed a complete response to treatment with no detectable variants. A different color represents each clone. (Source: ChrisMiller fishplot-R). (**B**) Clonal evolution plot illustrating the variant allele frequencies (VAFs) of somatic clones detected during longitudinal cfDNA monitoring (T0, T1, T2) under atezolizumab treatment.

At baseline (T0), before treatment initiation, high-depth cfDNA whole-exome sequencing identified a heterozygous germline mutation in the *WNK1* gene [*WNK1*, c.2172dup, p.(Pro725SerfsTer46)], on which three predominant expanding clones involving *KRAS* [c.38G>A, p.(Gly13Asp), VAF = 12%], *ATM* [c.5836C>T, p.(Gln1946Ter), VAF = 4.70%], and *NF1* [c.7954del, p.(Gln2652LysfsTer6), VAF = 3.50%] were arising, consistent with the molecular landscape detected at diagnosis (Figure 4A). Notably, cfDNA analysis confirmed the *KRAS* mutation previously identified in the FFPE somatic DNA, with a VAF of 48%.

After two months of therapy (T1), cfDNA analysis demonstrated a substantial reduction in the VAFs (Figure 4B) of all previously identified driver mutations, indicating a marked molecular response to *KRAS* [c.38G>A, p.(Gly13Asp), VAF = 2.89%], *ATM* (c.5836C>T, p.(Gln1946Ter), VAF = 2.25%), and *NF1* [c.7954del, p.(Gln2652LysfsTer6), VAF = 1.20%]. Concurrently, three novel low-frequency subclonal variants emerged [*TP53* c.437G>A, p.(Trp146Ter), VAF = 2.5%; *PTEN*, c.511C>A, p.(Gln171Lys), VAF = 0.17%; *FBXW7* c.1393C>T, p.(Arg465Cys), VAF = 0.22%] (Figure 4A), suggesting dynamic clonal remodeling under immunotherapeutic selective pressure.

At the third time point (T2), after five months of total treatment, cfDNA WES revealed complete molecular clearance of all previously detected clones, with no residual variants above the analytical threshold. This molecular complete remission was mirrored by radiological imaging, which documented progressive tumor shrinkage culminating in a disease-free status (Figure 3B).

Clonal dynamics across the three time points were visualized using an evolutionary fish plot (Figure 4A), clearly illustrating the contraction of dominant baseline clones (*KRAS*, *ATM*, *NF1*), the transient expansion of minor subclones (*TP53*, *PTEN*, *FBXW7*) at T1, and the eventual eradication of all detectable tumor-derived variants at T2. Imaging performed before T0 and after T1 and T2 was fully concordant with the molecular findings, further highlighting the capacity of longitudinal liquid biopsy to sensitively capture real-time tumor evolution and to anticipate clinical outcome, confirming durable responses to immunotherapy. 

Notably, patient 111/24 was the only one in our cohort lacking *TP53* mutations at baseline (T0). The subsequent emergence of a low-frequency *TP53* variant at T1 [p.(Trp146Ter), VAF = 2.5%] was transient and successfully cleared by T2, coinciding with complete molecular clearance, contrasting with the persistent, high-VAF *TP53* clones observed in the non-responding patients. 

### 2.3. Third Case

**Figure 5:**
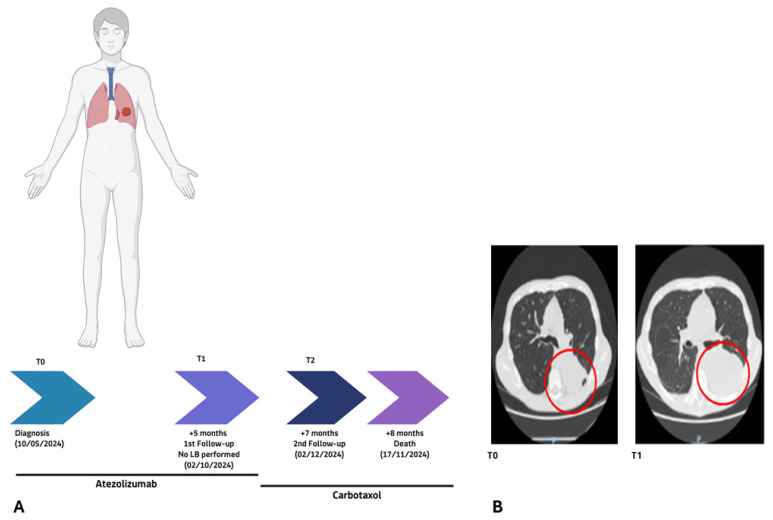
(**A**) Timeline illustrating the clinical course of patient 49/24. (**B**) 49/24 patient CT scans at baseline (T0) and at first follow-up (T1), confirming a no-response to immunotherapy. The red circle outlines the lung tumor mass. Anatomical orientations are indicated as P (posterior).

Patient 49/24 (male, 72 years old, PD-L1 90%) was initially treated with the immune checkpoint inhibitor atezolizumab (Figure 5A). Despite very high PD-L1 expression, which would normally predict a favorable response to immunotherapy, the patient showed no clinical or radiological benefit, as confirmed at the first follow-up (T1, Figure 5B).

Consequently, therapy was switched to carboplatin-paclitaxel (carbotaxol) chemotherapy, which achieved a molecular response (T2).

**Figure 6:**
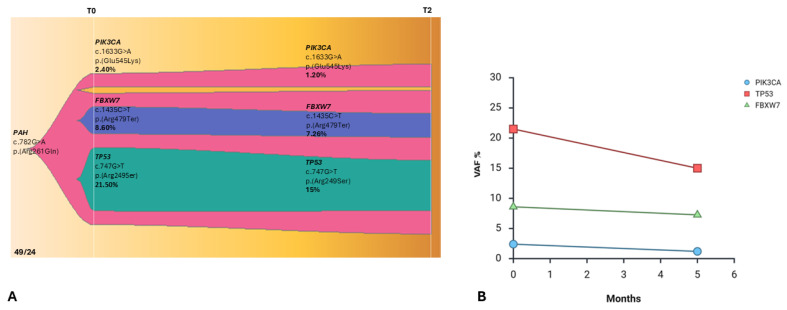
(**A**) Fishplot representation of clonal dynamics during treatment. Fishplot illustrating the temporal evolution of tumor clones in patient 49/24 across treatment timepoints. A different color represents each clone. (Source: ChrisMiller fishplot-R). (**B**) Variant allele frequency (VAF) dynamics of *TP53*, *PIK3CA*, and *FBXW7* clones in longitudinal analysis of cfDNA.

Genomic profiling through multi-point cfDNA WES revealed several variants that may help explain this unexpected lack of response to immunotherapy. Baseline cfDNA WES identified a high-VAF *TP53* mutation [c.747G>T, p.(Arg249Ser), VAF = 21.50%], alongside pathogenic variants in *PIK3CA* [c.1633G>A, p.(Glu545Lys), VAF = 2.40%] and *FBXW7* [c.1435C>T, p.(Arg479Ter), VAF = 8.60%]. At T2, following the therapeutic switch to carbotaxol chemotherapy, cfDNA analysis revealed an effective response in the previously identified clones, whose VAFs decreased to 15%, 1.20%, and 7.26%, respectively (Figure 6A,B). A germline mutation in the *PAH* gene, c.782G>A, p.(Arg261Gln), has been identified.

Longitudinal monitoring of cfDNA (Figure 6A) confirmed a decline in clonal burden of these variants after the switch to chemotherapy, highlighting the therapeutic efficacy of carbotaxol in this setting.

### 2.4. Fourth Case

**Figure 7:**
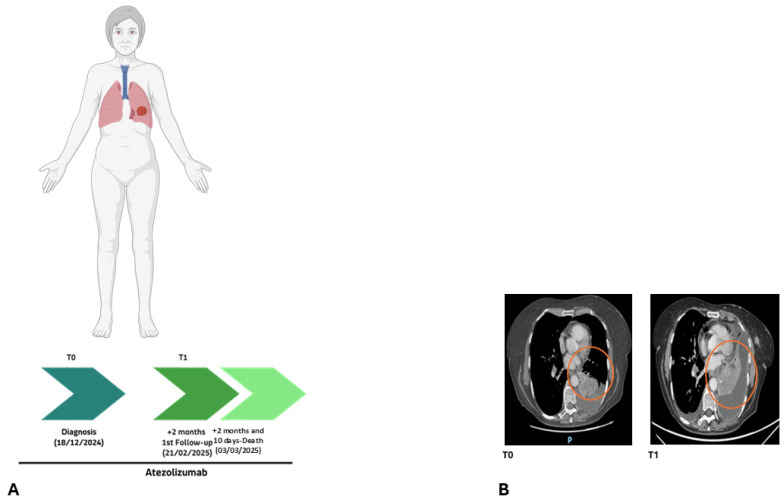
(**A**) Timeline illustrating the clinical course of Patient 151/24. (**B**) Patient 151/24 CT scans at baseline (T0) and at first follow-up (T1), confirming no-response to immunotherapy. The orange circle outlines the lung tumor mass, which was increasing in size. Anatomical orientations are indicated as P (posterior).

Comprehensive tumor molecular analysis was performed at baseline (T0) and after 2 months of treatment (T1) in patient 151/24, a 61-year-old female who was a PD-L1 high (90%) metastatic NSCLC non-responder to atezolizumab (Figure 7).

**Figure 8:**
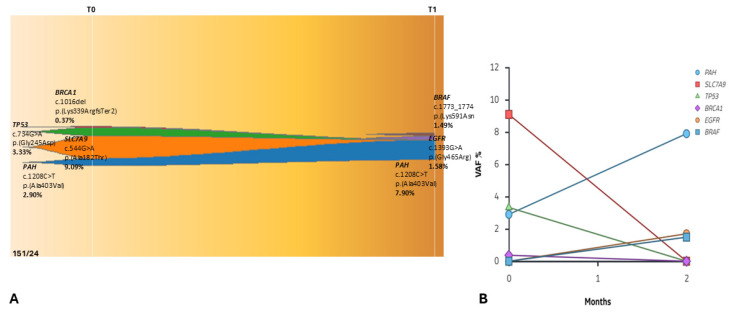
(**A**) Fishplot illustrating the longitudinal clonal dynamics of Patient 151/24 during immunotherapy. At baseline (T0), the genomic landscape was characterized by clonal variants in *PAH*, *SLC7A9*, *TP53*, and *BRCA1*. At the first follow-up (T1, 2 months), despite ongoing immunotherapy, the *PAH* variant increased in allele frequency. Additionally, new low-frequency alterations emerged in *EGFR* and *BRAF*, suggesting early subclonal diversification under treatment pressure. A different color represents each clone. (Source: ChrisMiller fishplot-R). (**B**) Variant allele frequency (VAF) dynamics of *PAH*, *SLC7A9*, *TP53*, *BRCA1*, *EGFR* and *BRAF* clones. At T0, the genomic landscape was characterized by clonal variants in *PAH* (c.1208C>T), *SLC7A9* (c.544G>A), *TP53* (c.734G>A), and *BRCA1* (c.1016del). At T1, an increase in the *PAH* variant VAF was observed. Additionally, novel subclonal alterations emerged in *EGFR* (c.1393G>A) and *BRAF* (c.1773_1774del).

At baseline (T0), the molecular landscape was characterized by clonal variants, including alterations in *PAH* [c.1208C>T, p.(Ala403Val), 2.90%], *SLC7A9* [c.544G>A, p.(Ala182Thr), 9.09%], *TP53* [c.734G>A, (p.Gly245Asp), 3.33%], and *BRCA1* [c.1016del, p.(Lys339ArgfsTer2), 0.37%] (Figure 8A).

At T1 (2 months later), despite immunotherapy exposure, the *PAH* variant also increased (7.90%). Notably, the genomic profile not only showed clonal persistence but also revealed early subclonal diversification. This was characterized by the emergence of novel alterations in *EGFR* (c.1393G>A, p.Gly465Arg; VAF = 1.58%) and *BRAF* (c.1773_1774del, p.Lys591AsnfsTer3; VAF = 1.49%), potentially reflecting early molecular evolution (Figure 8A,B).

Overall, the molecular profile demonstrates clonal persistence and expansion of pre-existing alterations rather than immune-mediated tumor clearance, with evidence of molecular progression. In this case, the liquid biopsy at T1 revealed an early molecular rebound of resistance clones. This molecular signal preceded the radiological documentation of progression (confirmed at T2, one month later, Figure 7B), illustrating a molecular lead time where the expansion of resistant clones was detectable in the cfDNA before becoming clinically evident on imaging.

## 3. Discussion

The longitudinal monitoring of cfDNA via high-depth WES in this case series provides critical insights into the evolutionary trajectories of metastatic lung adenocarcinoma under the selective pressure of immune checkpoint inhibitors (ICIs). Our findings underscore the clinical utility of liquid biopsy not only as a surrogate for tumor burden and evolution, but also as a highly sensitive tool for anticipating therapeutic resistance and molecular escape.

A central observation in our study is the correlation between VAF kinetics and clinical outcome. In Patient 111/24, the “molecular complete response”, characterized by the total clearance of *KRAS*, *ATM*, and *NF1* clones, accurately mirrored the radiological achievement of a disease-free status. Notably, this early molecular clearance (detectable at T1-T2) suggests a potential indicator for sustained clinical benefit from atezolizumab. This patient harbored mutations in *KRAS* (p.Gly13Asp) and *ATM* (p.Gln1946Ter). Notably, the identified *KRAS* G13D mutation was successfully cleared during treatment, a finding of interest given that different *KRAS* substitutions can exhibit distinct biological behaviors and responses to immunotherapy compared to the more frequent G12C variant. In Patient 111/24, we observed a molecular complete response in the cfDNA compartment, characterized by the total clearance of previously detectable plasma variants (from 48% in tissue to 0% in cfDNA at T2). The *ATM* is involved in DNA damage repair (DDR) [16], likely increasing the Tumor Mutational Burden (TMB) and paradoxically priming the tumor for extreme sensitivity to checkpoint inhibitors like atezolizumab [17].

Conversely, **Patient 66/24** illustrates the phenomenon of “molecular progression” preceding clinical decline. The rapid rebound of *TP53*, *NF1*, and *NOTCH2* VAFs at T2, coupled with the appearance of novel subclonal variants in *KIF1A* and *PTEN*, indicates the onset of acquired resistance. In this case, the molecular rebound provided a critical **lead time**, identifying the therapeutic failure synchronously with the development of cerebral metastases. This reinforces the ability of cfDNA to bypass the blood–brain barrier and capture the spatial and temporal heterogeneity of metastatic disease that single-tissue biopsies often miss.

The emergence of *PTEN* variants at T1/T2 in patients who initially responded (66/24 and 111/24) represents a classic example of acquired resistance. *PTEN* loss increases the expression of immunosuppressive cytokines, allowing the tumor to evade immune surveillance [18,19,20].

Again, inactivating variants in the *NF1* (Neurofibromin 1) gene, detected in both patients 66/24 (progression) and 111/24 (response), could act as a negative regulator of the RAS pathway. In Patient 66/24, the *NF1* VAF rebound at T2 (25.51%) was an early indicator of therapeutic failure and MAPK pathway reactivation [21,22]. This patient was also mutated in the *NOTCH2* gene (p.Gly1263Ter). *NOTCH2* mutations can alter cellular differentiation and stemness [23]. The clonal dynamics of *NOTCH2* perfectly mirrored the total tumor burden, confirming its utility as a monitoring marker.

However, there is a PD-L1 paradox. Our data highlight a significant clinical challenge: the poor correlation between high PD-L1 expression and durable ICI response in some patients. Despite PD-L1 levels ≥60%, patients 49/24 and 151/24 exhibited primary resistance to atezolizumab.

In **patient 49/24**, the presence of a *PIK3CA* (p.Glu545Lys) mutation and *FBXW7* alterations may explain this lack of response. The role of the PI3K/AKT/mTOR pathway in fostering an immunosuppressive tumor microenvironment has been extensively documented [24,25]. Mutations in the PI3K/AKT/mTOR pathway are known to modulate the tumor microenvironment and are often associated with an “immune-cold” phenotype, potentially negating the benefits of high PD-L1 expression [26,27,28,29,30]. Indeed, the *PIK3CA* (p.Glu545Lys) is a gain-of-function mutation that modulates the tumor microenvironment by reducing T-lymphocyte infiltration and explaining the lack of response despite 90% PD-L1 expression [28]. Specifically, the activation of this pathway leads to the upregulation of immunosuppressive cytokines and the exclusion of CD8+ T-cells, providing a mechanistic explanation for the primary resistance observed in Patient 49/24. Interestingly, the switch to carboplatin-paclitaxel induced a significant molecular response, demonstrating the utility of cfDNA in monitoring chemotherapy efficacy and suggesting that in the presence of specific co-mutations, traditional chemotherapy may remain superior to immunotherapy.

The emergence of new mutations during treatment, such as *EGFR* and *BRAF* variants in patient 151/24, reflects the rapid adaptive evolution of the tumor genome.

In **patient 151/24**, the persistence of *PAH* and SLC7A9 clones despite ICI therapy suggests a biologically resistant backbone. The detection of subclonal *EGFR* and *BRAF* alterations at T1, although at low VAFs, points toward **rapid adaptive evolution** as a mechanism of immune evasion. The presence of mutations in *PAH* and *BRCA1* in non-responding patients suggests a tumor microenvironment (TME) that is both metabolically and genetically hostile to T-cell activity [31,32]. While *PAH* is associated with hepatic metabolism, its alteration in lung cancer may indicate a significant **metabolic reprogramming**. In fact, *PAH* is responsible for the rate-limiting step in phenylalanine catabolism, and its dysfunction may lead to an accumulation of phenylalanine in the TME. The accumulation of phenylalanine or the dysregulation of its derivatives (such as tyrosine) can influence the synthesis of catecholamines and signaling molecules that modulate systemic and local inflammation [31,33,34]. In our data, the increase in *PAH* VAF during therapy, rising from 2.90% to 7.90%, suggests that this clone is not only resistant to atezolizumab but also possesses a selective advantage, serving as a marker of primary metabolic resistance. This ‘metabolic escape’ could create a hostile environment for immune cells, potentially explaining why these patients failed to respond despite high PD-L1 expression. Regarding the *BRCA1* mutation, theoretically, *BRCA1* defects should increase Tumor Mutational Burden (TMB) and, consequently, improve response to ICIs [35]. However, in Patient 151/24, the *BRCA1* mutation was subclonal and persisted despite treatment, suggesting that when such mutations are subclonal or not accompanied by loss of heterozygosity (LOH), they may contribute to “chronic genomic instability” rather than an immune-priming phenotype [36,37]. Furthermore, the *BRCA1* low VAF (0.37%) and the co-occurrence with *TP53* could nullify any potential immunogenic benefit. This state continuously generates novel variants, as evidenced by the emergence of *EGFR* and *BRAF* at T1, without producing sufficiently potent neoantigens to trigger an effective immune response. This results in an intratumoral heterogeneity that ultimately stifles the efficacy of immunotherapy.

The identification of germline variants (*WNK1* in patient 111/24 and *PAH* in patient 49/24) through WES adds another layer of complexity. While the specific role of these variants in lung cancer progression remains to be fully elucidated, the expansion of somatic “driver” clones (like *KRAS*) within a specific germline background may influence the overall fitness of the tumor and its interaction with the host immune system. However, due to their noncanonical role in NSCLC, their presence is reported for completeness of data due to incidental finding.

What we noticed in patients 49/24, 66/24 and 111/24 are the mutations in *TP53* (p.Val203Phe, p.Arg249Ser, p.Gly245Asp). Indeed, while *TP53* mutations are ubiquitous in NSCLC, their impact depends on their co-occurrence with other drivers. In non-responders, the persistence of high-VAF *TP53* clones served as a marker of genomic instability and poor prognosis. Furthermore, the expansion of somatic drivers on specific germline backgrounds (*WNK1* in patient 111/24, *PAH* in 49/24) adds a layer of complexity to tumor fitness and host immune interaction that warrants further investigation. The relationship between *TP53* status and immunotherapy efficacy remains complex. Patient 111/24, the only complete responder, was also the only patient lacking *TP53* mutations at diagnosis (T0). This contrasts with the non-responding cases, where the *TP53* mutations are present at baseline, contributing to a primary resistance to immunotherapy. This aligns with emerging evidence suggesting that baseline *TP53* mutations may act as a marker of higher genomic complexity and subclonal fitness, potentially hindering the efficacy of ICI monotherapy despite high PD-L1 expression. In our non-responders, the ‘*TP53*-mutant scaffold’ likely facilitated the rapid emergence of secondary escape mechanisms, such as *PTEN* or *PIK3CA* alterations. So, the baseline profile of cfDNA could be useful to identify patients suitable to benefit from monotherapy.

Finally, in patients 49/24 and 111/24, the *FBXW7* gene is a critical tumor suppressor that targets oncoproteins for degradation. According to systematic evidence, FBXW7 deficiency stabilizes oncoproteins such as MYC and Cyclin E, which in turn orchestrate an ‘immune-cold’ environment characterized by impaired antigen presentation and reduced recruitment of tumor-infiltrating lymphocytes [25]. Loss-of-function mutations (such as p.Arg479Ter) promote cell cycle progression and are frequently associated with an “immune-cold” phenotype and poor ICI efficacy [38,39,40,41]. Our finding of FBXW7 mutations in non-responding patients aligns with these comprehensive reviews, suggesting that FBXW7 status could serve as a potential negative predictor for ICI monotherapy.

Despite the high-depth molecular resolution provided by our longitudinal WES approach, several limitations of this study must be acknowledged. First, the small cohort size (*n* = 4) precludes formal statistical validation and limits the generalizability of our findings regarding specific ‘molecular signatures’ of resistance, which remain hypothesis-generating. Furthermore, the absence of paired sequencing of peripheral blood mononuclear cells (PBMCs) represents a technical constraint, as we cannot definitively exclude the influence of Clonal Hematopoiesis of Indeterminate Potential (CHIP). In order to address this, we focused on variants with high VAF or those previously documented as somatic drivers in the patient’s tissue biopsy.

The study nevertheless demonstrated that the use of high-depth WES analysis compensates for these limitations by providing a high-resolution, unbiased view of clonal dynamics that targeted NGS panels might overlook. By capturing low-frequency subclonal shifts and metabolic markers like *PAH*, our approach demonstrates that even a small case series can reveal complex mechanisms of immune evasion and metabolic reprogramming that are otherwise invisible to routine clinical diagnostics.

Our observation that cfDNA kinetics reflect radiological response is consistent with larger-scale analyses, such as those by Gandara et al. and results from the POPLAR and OAK trials, which established that early changes in circulating tumor DNA correlate with overall survival and treatment efficacy in NSCLC patients treated with ICIs [42]. By utilizing WES instead of targeted panels, our case series extends these observations, suggesting that even subclonal shifts can provide early warnings of treatment failure.

## 4. Conclusions

This study illustrates how longitudinal, high-depth cfDNA WES can provide detailed insights into the evolutionary landscape of metastatic NSCLC compared to baseline tissue biopsy or PD-L1 immunohistochemistry alone. While the initial tissue biopsy was limited by a targeted gene panel, the use of WES on plasma samples allowed us to track a broader spectrum of clonal and subclonal shifts over time. This longitudinal approach offers a higher temporal resolution of the mutational landscape compared to a static, single-point tissue assessment, providing insights into the molecular shifts occurring under therapeutic pressure. Our findings provide preliminary evidence that supports the following observations:

While PD-L1 expression remains the standard biomarker for ICI eligibility, it failed to predict clinical benefit in 50% of our cohort. In contrast, early cfDNA detection, specifically the disappearance of previously identified plasma driver clones at T1, served as an indicator of therapeutic success in our responder. Conversely, VAF rebound or persistence consistently preceded radiological progression, offering a critical window for clinical intervention.

We identified *TP53* mutations not merely as static markers of malignancy but as evolutionary transition variants. The persistence of high-VAF *TP53* clones provides a permissive genomic scaffold that facilitates “molecular escape,” allowing the tumor to rapidly acquire secondary resistance mutations (e.g., *PTEN*, *PIK3CA*) under the selective pressure of immunotherapy.

The failure of ICI in patients with high PD-L1 was associated with a genomic dynamism driven by subclonal *BRCA1* mutations and PAH-mediated metabolic reprogramming. These alterations create a tumor microenvironment that is both genetically heterogeneous and metabolically hostile to T-lymphocyte activity, effectively bypassing PD-L1 blockade.

In our cohort, the rapid molecular response observed after switching to platinum-based chemotherapy in patients with *PIK3CA* or *FBXW7* co-mutations illustrates how longitudinal cfDNA monitoring may identify clones associated with a resistant phenotype early in the treatment course. While these findings are limited to individual cases, they align with broader evidence suggesting that activation of the PI3K pathway and loss of FBXW7 are recurrent features of immune evasion in NSCLC [24,41]. Our data supports the potential utility of cfDNA to monitor these high-risk molecular profiles early in the clinical course. This molecular compass could enable clinicians to pivot to chemotherapy or targeted agents months before radiological failure is documented.

In summary, integrating multi-point liquid biopsy into the clinical workflow offers a real-time map of the tumor’s life cycle. This approach allows for the identification of patients trapped in a biologically resistant phenotype, paving the way for more personalized and dynamic therapeutic strategies that move beyond the limitations of static biomarkers in metastatic lung adenocarcinoma.

## Figures and Tables

**Table 1:** Characteristics of metastatic NSCLC patients treated with first-line atezolizumab.

Patient ID	Age at Diagnosis	Sex	Smoking Status	Histology and Stage	PD-L1 Expression	Initial Systemic Therapy	Response	Alive	Available LB Timepoints	Tissue Molecular Analysis
66/24	55	F	Current Smoker	Metastatic Adenocarcinoma	60%	Atezolizumab monotherapy	Response and resistance	No	T0, T1, T2	*ALK* negative; *EGFR* negative
111/24	68	M	Current Smoker	Metastatic Adenocarcinoma	90%	Atezolizumab monotherapy	Responder	Yes	T0, T1, T2	*KRAS* (p.Gly13Asp) 48%
49/24	72	M	Current Smoker	Metastatic Adenocarcinoma	90%	Atezolizumab monotherapy, switched to carbotaxol	Non-responder	No	T0, T2	NA
151/24	69	F	Former Smoker	Metastatic Adenocarcinoma	90%	Atezolizumab monotherapy (later discontinued), switched to carbo + pemetrexed	Non-responder	No	T0, T1	*ERBB2* (p.Ile655Val) 24%

NA = Not Available.

## Data Availability

The raw data supporting the conclusions of this article will be made available by the authors on request.

## Supplementary Materials

The following supporting information can be downloaded at: https://www.mdpi.com/article/10.3390/ijms27072947/s1.

## Abbreviations

The following abbreviations are used in this manuscript:

ICI	Immune Checkpoint Inhibitor
PD-L1	Programmed Death-Ligand 1
NSCLC	Non-Small Cell Lung Cancer
cfDNA	Cell-free DNA
WES	Whole-Exome Sequencing
LC	Lung Cancer
AOUS	Azienda Ospedaliero-Universitaria Senese
FFPE	Formalin-Fixed Paraffin-Embedded
ICI software	Illumina Connected Insights software
MAF	Minor Allele Frequency
VAF	Variant Allele Frequency
DDR	DNA Damage Repair
TMB	Tumor Mutational Burden
