# Supplementary material for: Longitudinal Whole-Exome Sequencing of Cell-Free DNA Reveals Molecular Evolution and Heterogeneous Clinical Outcomes in PD-L1 Stratified Advanced NSCLC Adenocarcinoma Patients Treated with Atezolizumab
Source: Int J Mol Sci. 2026 Mar 24;27(7):2947. doi: 10.3390/ijms27072947 (PMC13073845; doi:10.3390/ijms27072947)
Supplement: Supplementary file 1 [file ijms-27-02947-s001.zip › ijms-4195129-supplementary.pdf]

Supplementary Methods: Longitudinal cfDNA Whole-Exome Sequencing and Bioinformatic Workflow

1. Patient enrollment

Four patients diagnosed with advanced adenocarcinoma (ADCA) of lung cancer were enrolled at the Medical Oncology Unit of the *Azienda Ospedaliero-Universitaria Senese (AOUS)* in Siena, Italy, between May 2024 and December 2024. Patients with early-stage LC were excluded, as they were candidates for standard curative treatments. The study was approved by the Institutional Ethics Committee (protocol n. 24962). All participants provided written informed consent prior to inclusion. Clinical data, including cancer family history, as well as peripheral blood samples, were collected at diagnosis, after treatment initiation, and/or during follow-up (1–3 samples per patient). The main clinical characteristics of the patient cohort and overall survival (OS) data are summarized in Table S1.

2. Sample collection and processing

For each patient, 10 mL of peripheral blood was collected into PAXgene Blood ccfDNA Tube (Qiagen). Plasma separation was achieved through a two-step centrifugation protocol, consisting of an initial centrifugation at 1900 × g for 20 minutes followed by a second centrifugation at 1900 × g for 15 minutes. Plasma was then carefully aliquoted and stored at –80°C until further processing.

Cell-free DNA (cfDNA) was extracted from 4 mL of plasma using the MagMAX Cell-Free Total Nucleic Acid Isolation Kit (Thermo Fisher Scientific, Waltham, MA, USA), according to the manufacturer's instructions. cfDNA concentration was quantified using the Qubit fluorometric assay (Invitrogen, Carlsbad, CA, USA) with the Qubit dsDNA HS Assay Kit, while fragment size distribution and quality were assessed using the High Sensitivity D1000 ScreenTape on the TapeStation 4150 System (Agilent Technologies, Santa Clara, CA, USA).

3. Library preparation and cfDNA whole-exome sequencing

For library preparation, 20 ng of cfDNA per sample were processed using the Illumina Cell-Free DNA Prep with Enrichment Kit (Illumina, San Diego, CA, USA). Prepared libraries were sequenced on the NovaSeq X Plus Sequencing System (Illumina platform) using a 25B Illumina flow cell, with four samples loaded per lane. Sequencing was performed in paired-end mode at high depth, generating approximately 50–72 billion reads per run and enabling whole-exome sequencing (WES) analysis of circulating cfDNA.

4. FFPE gDNA analysis

For patients whose FFPE (Formalin- Fixed Paraffin-Embedded) was available after biopsy or surgery, the FFPE gDNA has been extracted by using MagCore® Genomic DNA FFPE One-Step Kit for MagCore® System (Diatech Pharmacogenetics srl, Jesi, Ancona, Italy) [15]. The libraries of gDNA were prepared using a custom gene panel and sequenced on Illumina NextSeq 550.

5. Bioinformatic analysis

Secondary data analysis was performed using Illumina DRAGEN Enrichment software, achieving mean sequencing coverages ranging from 167X to 730X. Raw FASTQ files were aligned to the human reference genome GRCh38, and variant calling was conducted using Illumina Connected Insights (ICI) software.

Variant annotation and clinical classification were carried out using multiple curated databases, including JAX-CKB™, OncoKB™, COSMIC, CIViC, ClinVar, and OMIM. Only exonic, non-synonymous variants were retained for downstream analyses. Variants with a minor allele frequency (MAF) >1% in public population databases were excluded. Somatic tumor variants were defined by a variant allele frequency (VAF) >1%, while variants with VAFs between 50–60% or >95% were classified as *putative germline*. Somatic variants were prioritized within a VAF range from 1% to 39% to focus on tumor-derived clonal and subclonal dynamics. In parallel with Illumina Connected Insights (ICI), an independent in-house bioinformatic pipeline was applied to the raw FASTQ files to compare analytical performance and provide additional validation. Reads were quality-controlled and trimmed, aligned to GRCh38 using BWA-MEM, and processed for duplicate marking and base quality recalibration. Somatic variants were identified using GATK Mutect2 with standard filtering, retaining high-confidence coding variants (>95% reduction of initial calls), and annotated with OpenCRAVAT. Results were systematically compared with ICI outputs to assess concordance. Longitudinal clonal evolution was visualized using fish plots generated with the Chrismiller R package.

Supplementary Table S1. **Molecular variants found at each time point for each patient.**

66/24<br>T0	***66/24<br>T1***	***66/24<br>T2***	***111/24<br>T0***	***111/24<br>T1***	***111/24<br>T2***	***49/24<br>T0***	***49/24<br>T2***	***151/24<br>T0***	***151/24<br>T1***
TP53<br>c.607_609delinsTTT<br>p.(Val203Phe)<br>21.55%	***TP53***<br>c.607_609delinsTTT<br>p.(Val203Phe)<br>10.19%	***TP53***<br>c.607_609delinsTTT<br>p.(Val203Phe)<br>26.70%	***WNK1***<br>c.2172dup<br>p.(Pro725SerfsTer46)<br>49.60%	***WNK1***<br>c.2172dup<br>p.(Pro725SerfsTer46)<br>42%	***WNK1***<br>c.2172dup<br>p.(Pro725SerfsTer46)<br>43%	***PAH***<br>c.782G>A<br>p.(Arg261Gln)<br>52%	***PAH***<br>c.782G>A<br>p.(Arg261Gln)<br>44.40%	***PAH***<br>c.1208C>T<br>p.(Ala403Val)<br>2.90%	***PAH***<br>c.1208C>T<br>p.(Ala403Val)<br>7.90%
NF1<br>c.5458C>T<br>p.(Gln1820Ter)<br>17.62%	***NF1***<br>c.5458C>T<br>p.(Gln1820Ter)<br>4.17%	***NF1***<br>c.5458C>T<br>p.(Gln1820Ter)<br>25.51%	***KRAS***<br>c.38G>A<br>p.(Gly13Asp)<br>12%	***KRAS***<br>c.38G>A<br>p.(Gly13Asp)<br>2.89%		***TP53***<br>c.747G>T<br>p.(Arg249Ser)<br>21.50%	***TP53***<br>c.747G>T<br>p.(Arg249Ser)<br>15%	***TP53***<br>c.734G>A<br>p.(Gly245Asp)<br>3.33%	***EGFR***<br>c.1393G>A<br>p.(Gly465Arg)<br>1.58%
NOTCH2<br>c.3787G>T<br>p.(Gly1263Ter)<br>15.27%	***NOTCH2***<br>c.3787G>T<br>p.(Gly1263Ter)<br>6.29%	***NOTCH2***<br>c.3787G>T<br>p.(Gly1263Ter)<br>17.91%	***ATM***<br>c.5836C>T<br>p.(Gln1946Ter)<br>4.70%	***ATM***<br>c.5836C>T<br>p.(Gln1946Ter)<br>2.25%		***FBXW7***<br>c.1435C>T<br>p.(Arg479Ter)<br>8.60%	***FBXW7***<br>c.1435C>T<br>p.(Arg479Ter)<br>7.26%	***SLC7A9***<br>c.544G>A<br>p.(Ala182Thr)<br>9.09%	***BRAF***<br>c.1773_1774del<br>p.(Lys591AsnfsTer3)<br>1.49%
		KIF1A<br>c.317C>A<br>p.(Thr106Asn)<br>12.50%	***NF1***<br>c.7954del<br>p.(Gln2652LysfsTer6)<br>3.50%	***NF1***<br>c.7954del<br>p.(Gln2652LysfsTer6)<br>1.2%		***PIK3CA***<br>c.1633G>A<br>p.(Glu545Lys)<br>2.40%	***PIK3CA***<br>c.1633G>A<br>p.(Glu545Lys)<br>1.20%		
		PTEN<br>c.71A>G<br>p.(Asp24Gly)<br>0.70%		***TP53***<br>c.437G>A<br>p.(Trp146Ter)<br>2.50%					
				PTEN<br>c.511C>A<br>p.(Gln171Lys)<br>0.17%					
				FBXW7<br>c.1393C>T<br>p.(Arg465Cys)<br>0.22%					
